# Supplementary material for: Substance Use in the Transgender Population: A Meta-Analysis
Source: Brain Sci. 2022 Mar 10;12(3):366. doi: 10.3390/brainsci12030366 (PMC8945921; doi:10.3390/brainsci12030366)
Supplement: Supplementary file 1 [file brainsci-12-00366-s001.zip › Figure S3.pdf]

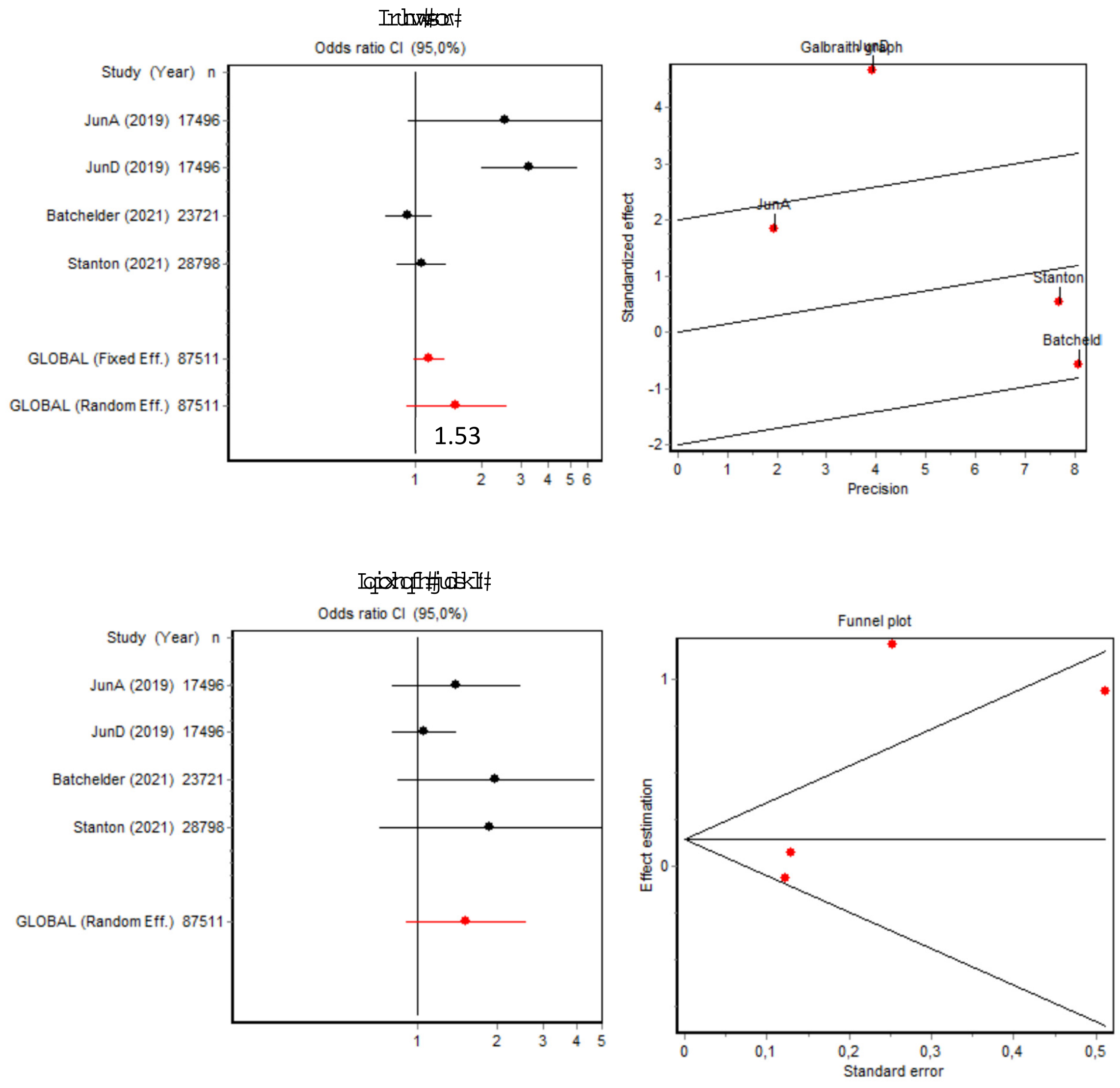

Figure S3. Forest plot, Galbraith graphic, influence graphic, and funnel plot for current substance use disorder. A: Abuse; D: Dependency.
